# Alpha Synuclein Connects the Gut-Brain Axis in Parkinson’s Disease Patients – A View on Clinical Aspects, Cellular Pathology and Analytical Methodology

**DOI:** 10.3389/fcell.2020.573696

**Published:** 2020-09-08

**Authors:** Eva Schaeffer, Annika Kluge, Martina Böttner, Friederike Zunke, Francois Cossais, Daniela Berg, Philipp Arnold

**Affiliations:** ^1^Department of Neurology, Christian-Albrechts-University of Kiel, Kiel, Germany; ^2^Institute of Anatomy, Christian-Albrechts-University of Kiel, Kiel, Germany; ^3^Biochemical Institute, Christian-Albrechts-Universität zu Kiel, Kiel, Germany; ^4^Department of Neurodegeneration, Hertie Institute for Clinical Brain Research, University of Tübingen, Tübingen, Germany; ^5^MSH Medical School Hamburg, Hamburg, Germany

**Keywords:** Parkinson’s disease, alpha synuclein, ultra-structural analysis, gastrointestinal tract, gut-brain axis

## Abstract

Parkinson’s disease (PD) is marked by different kinds of pathological features, one hallmark is the aggregation of α-synuclein (aSyn). The development of aSyn pathology in the substantia nigra is associated to the manifestation of motor deficits at the time of diagnosis. However, most of the patients suffer additionally from non-motor symptoms, which may occur already in the prodromal phase of the disease years before PD is diagnosed. Many of these symptoms manifest in the gastrointestinal system (GIT) and some data suggest a potential link to the occurrence of pathological aSyn forms within the GIT. These clinical and pathological findings lead to the idea of a gut-brain route of aSyn pathology in PD. The identification of pathological aSyn in the intestinal system, e.g., by GIT biopsies, is therefore of highest interest for early diagnosis and early intervention in the phase of formation and propagation of aSyn. However, reliable methods to discriminate between physiological and pathological forms of enteral aSyn on the cellular and biochemical level are still missing. Moreover, a better understanding of the physiological function of aSyn within the GIT as well as its structure and pathological aggregation pathways are crucial to understand its role within the enteric nervous system and its spreading from the gut to the brain. In this review, we summarize clinical manifestations of PD in the GIT, and discuss biochemical findings from enteral biopsies. The relevance of pathological aSyn forms, their connection to the gut-brain axis and new developments to identify pathologic forms of aSyn by structural features are critically reviewed.

## Introduction

Alpha synuclein (aSyn) is a small protein that consists of 140 amino acids and is primarily found as a monomer in the cellular cytosol. Here, it plays a role in synaptic plasticity and interacts with presynaptic vesicles (Lashuel et al., 2013). Under pathological conditions, aSyn monomers aggregate and form amyloids, which have been shown to exert neurotoxic properties (Lashuel et al., 2013; Riederer et al., 2019). These aSyn amyloids can be found in patients suffering from neurodegenerative disorders collectively known as synucleinopathies, comprising Parkinson’s disease (PD), multiple system atrophy (MSA) and dementia with Lewy bodies (DLB) (Spillantini et al., 1997; Goedert et al., 2017; Riederer et al., 2019).

In PD patients, aSyn aggregates form Lewy bodies and Lewy neurites and are detected in post-mortem brains (Spillantini et al., 1997; Braak et al., 2003). Dopaminergic neurons residing in the substantia nigra pars compacta are the most affected central nervous system (CNS) cell population during the disease course. A loss of these neurons leads to a reduction of the neurotransmitter dopamine, thereby influencing feedback loops within the basal ganglia, resulting in clinical symptoms including rigidity, bradykinesia and tremor (Kalia and Lang, 2015). However, in recent years, aSyn and its aggregates were also found in the gastro-intestinal tract (GIT) of PD patients and symptoms outside the CNS were described including obstipation and reduced peristalsis (Braak et al., 2006; Postuma et al., 2012; Beach et al., 2016). This gave rise to the hypothesis that manifestations in the brain and in the gut are two sides of the same coin. Some researchers even favor the idea that PD pathology can spread from the gut to the brain and/or vice versa (Braak et al., 2006; Breen et al., 2019; Leclair-Visonneau et al., 2020) and a transmission of aSyn pathology via the vagal nerve was suggested at least in an animal model (Kim S. et al., 2019; Challis et al., 2020).

**FIGURE 1:**
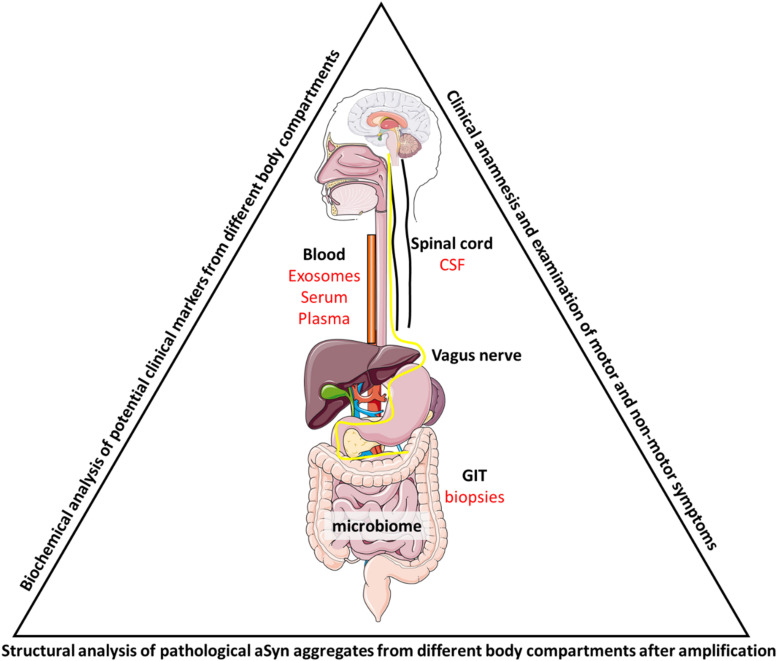
Summarizing figure. To analyze PD progression during the disease course, patient samples have to be taken from appropriate sources to allow multiple sampling of the same patient. These include biopsies (e.g., rectal biopsies during colonoscopy for cancer screening), serum, plasma or exosomes from blood samples and CSF from lumbar puncture. Biochemical and structural analysis could then link the different aSyn species to different cell types either via structure determination after (multiple rounds of) amplification by PMCA or through the purification of cell-type specific exosomes. Identification of markers that discriminate patients from each other or patients from control individuals could then be mirrored into clinical practice and be linked to patient outcome. Also, the gut microbiome has been shown to be involved in disease pathology and further studies will have to untangle the relationship between PD, aSyn aggregation and gut dysbiosis.

At the cellular level, different compartments are of interest when looking at aSyn homeostasis and pathological aggregation pathways. aSyn can be degraded via the proteasomal or via the lysosomal system, where different cathepsins have been proposed to mediate aSyn degradation (Webb et al., 2003; Sevlever et al., 2008; Cullen et al., 2009; Mak et al., 2010; Xilouri et al., 2013). Elevation of the physiological aSyn level via increased production (e.g., gene duplication or triplication) or reduced degradation leads to an accumulation of intracellular aSyn. This accumulation results in the aggregation of the aSyn protein leading to the formation of different conformers, including toxic and non-toxic oligomers, tetramers and fibrils (Bartels et al., 2011; Lashuel et al., 2013; Wong and Krainc, 2017; Zunke et al., 2018), with β-sheet rich fibrils being the endpoint of the aggregation process (Cremades et al., 2012; Lashuel et al., 2013). Aggregated aSyn was also shown to escape from the lysosome to the cytosol via still not well understood pathways (Jiang et al., 2017; Karpowicz et al., 2019). Moreover, aSyn conformers can be released from the entire cell, most importantly not only from dying cells, but also within CNS-derived extracellular vesicles (exosomes), which can be found in the blood and CSF (Shi et al., 2014; Fussi et al., 2018; Parnetti et al., 2019; Jiang et al., 2020; Figure 1). These peripheral aSyn species can be taken up by cells for degradation, but have also be shown to act as aggregation seeds, leading to an accelerated aggregation of toxic aSyn species, explaining the spreading and seeding capacity of the protein (Wood et al., 1999; Luk et al., 2009, 2012; Rey et al., 2019).

In this review, we highlight different aspects of aSyn pathology in PD patients, with an emphasis on the GIT. We will cover clinical aspects, look at basic findings that connect the GIT to PD development, discuss the role of enteral aSyn as a biomarker in PD, and evaluate methods to differentiate aSyn species on the cellular and structural level. At the end, we will conclude how these different research areas could be brought together for a better understanding of especially early PD stages.

## ASYN in the Gastro-Intestinal System: Physiological Expression and Function

While aSyn is well known for its pathological features, its physiological expression and function in the CNS as well as in the enteric nervous system (ENS) is not fully understood yet.

Physiological aSyn expression, including its phosphorylated forms, has been observed not only in the CNS, but also other peripheral tissues including the GIT (Bottner et al., 2012; Visanji et al., 2015; Barrenschee et al., 2017). Until now, our understanding of the physiological functions of aSyn in the CNS is incomplete, but even less is known about its impact and properties within the ENS. Whereas aSyn aggregates were first reported in vasoactive intestinal peptide (VIP)-positive neurons in humans (Wakabayashi et al., 1990), expression in both, VIP-positive (Chen et al., 2018) and cholinergic enteric neurons, potentially influencing cholinergic synaptic transmission, has been described in rodents (Wang et al., 2012; Swaminathan et al., 2019). A morphological and a co-localizing study gave evidence that aSyn is physiologically associated to the synaptic vesicle apparatus of enteric neurons (Böttner et al., 2015). Expression of aSyn is regulated by cyclic AMP (Paillusson et al., 2010) and aSyn secretion is activity-dependent in enteric neurons (Paillusson et al., 2012). Despite these interesting first findings, there is still a crucial lack of information regarding aSyn regulation and functions in the GIT under physiological conditions.

## Gastrointestinal Symptoms in PD: Indications for a Start of ASYN Pathology in the Gut

The occurrence of cardinal motor symptoms in PD is accompanied by aSyn aggregation in the substantia nigra, leading to the conclusion that aSyn pathology plays a pivotal role in PD. However, the disease is also characterized by a variety of non-motor symptoms. Amongst them, symptoms of impaired gastrointestinal function are very common, with approximately 80% of PD patients being affected by at least one gastrointestinal symptom during the course of the disease, indicating an additional (aSyn) pathology in the GIT (Edwards et al., 1991; Cersosimo et al., 2013). These symptoms do not only play an important role in the disease burden for the patient, but also affect treatment of the disease, as medication is less regularly absorbed. Additionally, gastrointestinal affection also gives an important insight into underlying pathological mechanisms and etiologic factors.

Already in his first description of PD, James Parkinson mentioned the severe symptom of reduced bowel movements with a frequent need for pharmacological or even physical intervention (Palacios-Sanchez et al., 2017). Since then, several gastrointestinal symptoms have been identified, affecting nearly all parts of the gastrointestinal system starting from the salivary glands and esophagus (hypersalivation and dysphagia), including the stomach (gastroparesis/delayed gastric emptying), the small intestine and colon (constipation) and the rectum and anus (anorectal/defecatory dysfunction) (Pfeiffer, 2018). These symptoms reveal that besides neurons of the CNS, also large parts of the peripheral nervous system (PNS), including the parasympathetic (Nervus vagus), the sympathic (Nervi splanchnici) nervous system and the ENS are affected in PD.

The individual description of gastrointestinal symptoms by PD patients has been complemented by a variety of objective imaging techniques. Methods to quantify functional impairments of the gastrointestinal system include esophageal and gastric scintigraphy to display dysphagia and delayed gastric emptying (Hardoff et al., 2001; Potulska et al., 2003), MRI (magnetic resonance imaging) techniques to measure colonic enlargement (Knudsen et al., 2017a) and Donepezil PET to display cholinergic denervation of the gut (Gjerloff et al., 2015). Recent studies with ingestible capsule systems using the radio opaque marker technique or SPECT/CT confirmed a high prevalence of reduced intestinal transit time in PD (Sakakibara et al., 2003; Dutkiewicz et al., 2015; Knudsen et al., 2017b). Interestingly, objective imaging techniques were found to be more sensitive than subjective ratings of patients (Knudsen et al., 2017a). Additionally, sonographic studies confirm a direct impairment of the vagal nerve by displaying nerve atrophy with high-resolution ultrasound (Pelz et al., 2018; Walter et al., 2018).

Importantly, clinical symptoms of gastrointestinal dysfunction, particularly constipation, are not only an expression of advanced disease, but occur also in very early phases, often years before the onset of the typical motor symptoms. The phase of ongoing neurodegeneration preceding the clinical diagnosis is defined as the prodromal phase of PD, with constipation being one of the most important prodromal symptoms. Findings of large retro- and prospective studies showed that constipation is also one of the earliest prodromal symptoms, occurring up to twenty years before cardinal motor signs manifest (Abbott et al., 2001; Gao et al., 2011; Ross et al., 2012). This clinical observation, together with the breakthrough pathological findings of Braak et al. (2003), who histologically described ascending aSyn pathology, led to the hypothesis that pathology in PD may start in and spread from the gut at least in a subpopulation of patients (Figure 1). The Braak staging system proposed a first conceptual link between the presence of aSyn in the GIT and its spreading via the vagal nerve to its dorsal motor nucleus (DMV) in the brainstem. This presumed ascending propagation of aSyn pathology via the vagal nerve was clinically reassured by findings of lower incidence of PD in individuals receiving vagotomy (Svensson et al., 2015). However, Braak et al. already assumed in their dual-hit hypothesis that pathology does not always follow the gut-brain route and proposed a secondary propagation path via the olfactory bulb (Hawkes et al., 2007). Indeed, in recent years, evidence is slowly emerging that the gut-brain-propagation in PD may define a subtype of the disease, with distinct underlying pathological mechanism and clinical phenotypes (Figure 1). One approach to identify subtypes of PD already in the prodromal phase is the clinical identification of different non-motor symptom clusters (Marras and Chaudhuri, 2016), not only comprising gastrointestinal dysfunction, but also the occurrence of REM sleep behavior disorder (RBD). This parasomnia is associated with neuronal damage in the brainstem and individuals with idiopathic RBD have an over 80% risk of developing PD in the future (Romenets et al., 2012; Iranzo et al., 2014; Postuma et al., 2019).

Knudsen et al. (2018) were recently able to show in RBD patients both, cholinergic denervation of the gut using Donepezil PET and cardiac sympathetic denervation using metaiodobenzylguanidine (MIBG) scintigraphy. Additionally, the lower brainstem (as shown by neuromelanin-MRI) but not (yet) the nigrostriatal system (displayed using F-DOPA PET) were affected by the neurodegenerative process.

Taken together, affection of the GIT is not only relevant for patients and the basis for clinical and imaging markers, but may also serve as a source for the identification of molecular markers within the GIT, which is far easier to access than the CNS. It is therefore of highest interest to understand the role of pathological aSyn as potential molecular marker in the GIT.

## ASYN in the Gastro-Intestinal System: Pathological Implications

First characterizations of aSyn aggregates in the ENS of PD patients has been performed on autopsied specimens in the late 80’s by the group of Wakabayashi et al. (1988). Twenty years later, Lebouvier and co-workers characterized the presence of phosphorylated aSyn in routine colorectal biopsies of PD patients (Lebouvier et al., 2008). Since then, an increasing number of studies has aimed at evaluating the use of aSyn detection in the GIT as a potential biomarker for PD development (Tsukita et al., 2019). It is important to note that aSyn was not only detected in the colon and rectum, but also in other parts of the gastrointestinal system, including the salivary glands, lower parts of the esophagus and the stomach, corresponding to the above-mentioned clinical manifestations (Fayyad et al., 2019). In fact, many studies confirmed a rostro-caudal gradient of aSyn aggregates, questioning the colon and rectum as most suitable regions for biopsy studies (Braak et al., 2006; Beach et al., 2010; Adler et al., 2014). However, apart from the easy accessibility (e.g., in routine colonoscopies), the idea of dysbiosis as potential trigger of neurodegeneration still argues for colorectal biopsies to detect pathological changes of PD at the very beginning (Pietrucci et al., 2019; Nishiwaki et al., 2020).

One major issue regarding the development of aSyn as a biomarker for PD suitable for use in GIT-samples has been the difficulty to discriminate between native and pathologic forms of this protein in intestinal tissues. Indeed, native or phosphorylated forms of aSyn, as well as proteinase-K-resistant aggregates of aSyn have been detected in intestinal samples of PD patients, but no clear consensus has yet emerged about a detection method of pathological aSyn aggregates in the GIT (Beach et al., 2018). Despite these controversies, two independent studies reported increased aSyn deposits in early and prodromal PD patients (Shannon et al., 2012; Stokholm et al., 2016). One study performed by the multi-center Systemic Synuclein Sampling Study (S4) consortium demonstrated that aSyn expression patterns in the sigmoid colon can be used to distinguish between PD and healthy controls by trained neuropathologists with a sensitivity and specificity of almost 100% on a small cohort of 3 PD patients (Beach et al., 2018). Although globally encouraging, none of the methods published so far has reached sufficient specificity, sensitivity or reproducibility to serve as the basis for a potent biomarker for clinical diagnosis. On the contrary: characterization of aSyn pathological aggregates in human intestinal tissues is still the focus of vivid debates in literature (Schneider et al., 2016; Scheperjans et al., 2018; Bu et al., 2019; Tsukita et al., 2019).

Apart from intestinal tissues, the involvement of the vagal nerve in the spreading of PD from the gut to the brain has gained further support from animal models, following the above-mentioned hypothesis of Braak et al.^,^ For instance, either human pathologic or human recombinant aSyn was detected in the DMV of rats six days after its first introduction in the GIT (Holmqvist et al., 2014). Similar propagation of pathological aSyn to the CNS after injection in the GIT was confirmed in recent studies (Kim J. Y. et al., 2019; Van Den Berge et al., 2019; Challis et al., 2020). In these studies, injection of aSyn fibrils in the GIT even led to the development of PD-like symptoms. Both, spreading of aSyn accumulation and resulting symptoms were shown to depend on the integrity of the vagal nerve, as well as on the expression of endogenous aSyn in these models (Kim S. et al., 2019; Figure 1). Additionally, it was shown that injection of aSyn fibrils in the duodenal mucosa led to pathological aggregations of aSyn within the ENS, accompanied by intestinal inflammation, altered intestinal motility and further propagation of the disease to the CNS in aged mice (Challis et al., 2020). Interestingly, neuronal GBA1 [encoding for β-glucocerebrosidase (GCase)] overexpression partially rescued the induced aSyn accumulation and GIT dysfunction observed in these mice, indicating that GCase may play an important role in the regulation of aSyn life-cycle and pathological aggregation in enteric neurons (Challis et al., 2020). Here, the enzymatic substrate of GCase, β-glucosylceramide, might play an important role as it was shown to stabilize pathologic forms of aSyn (Zunke et al., 2018). Additionally, caudo-rostral propagation of aSyn was detected in rats after expression of human aSyn in the medulla oblongata via adeno-associated viral vectors toward the pons, midbrain and forebrain (Ulusoy et al., 2013).

Until now, little is known about the mechanisms regulating the formation of pathological aSyn species in the GIT. Proteinase-K-resistant aSyn aggregates were also observed in the vermiform appendix of healthy humans (Killinger et al., 2018). Appendectomy was associated with a decreased risk to develop PD, suggesting that pathological forms of aSyn in the vermiform appendix may contribute to spreading of the disease (Killinger et al., 2018). Further, recent evidence indicates that aSyn regulation and inflammatory processes are remarkably linked to each other, although a clear picture about their mutual imbrication is still missing [see for reviews (Rolli-Derkinderen et al., 2019; Tan et al., 2020)]. For instance, it was shown that a common infection in the human GIT results in an upregulation of aSyn expression in enteric neurons that positively correlated with the degree of acute and chronic inflammation in the intestinal wall and that monomeric and oligomeric aSyn have chemoattractant activity causing the migration of immune cells (Stolzenberg et al., 2017). Additionally, aSyn expression, but not its pathologic aggregation, is increased in the ENS of patients with Crohn’s disease (Prigent et al., 2019b) and inoculation of aSyn fibrils in the GIT is associated with an increased expression of inflammatory mediators in intestinal tissues (Challis et al., 2020). However, by contrast, it was seen that acute inflammatory stress inhibits aSyn expression in primary enteric neurons (Prigent et al., 2019a). Interestingly, inflammatory parameters, pathological aSyn aggregation and motor deficits were demonstrated to be regulated by microbiota in aSyn overexpressing mice (Sampson et al., 2016). The same group demonstrated that curly fibers derived from the bacterial amyloid CsgA, regulate not only the pathological aggregation of aSyn, but also the further development of intestinal and motor symptoms and inflammation in a similar mouse model (Sampson et al., 2020). Expression of aSyn in the intestinal mucosa does not seem to be limited to the ENS, but was also found in enteroendocrine cells (EECs) and in transit between enteric neurons and EECs through their neuropods (Chandra et al., 2017). Although the contribution of ECCs to PD pathology remains largely unclear, these cells are also interconnected to vagal efferents (Kaelberer et al., 2018), offering a direct potential road for the propagation of PD pathology from the intestinal mucosa to the brain, which may even bypass the ENS.

## Structural Aspects of ASYN Pathology

As described in the previous parts, there are good arguments that favor the onset and manifestation of PD in the GIT at least in a subpopulation of patients. The challenge is still the discrimination of patients and controls utilizing GIT-derived samples and an aSyn specific detection system. From a clinical but also cell-biological and biochemical view there are some arguments that favor GIT-specimens (especially from the colon) over other described sources as e.g., skin or blood (Fayyad et al., 2019; Ma et al., 2019). Interestingly, mice overexpressing human aSyn in neuronal cells (CNS and ENS), exhibit intestinal dysfunction besides the motor impairments (Chesselet et al., 2012). This indicates that aSyn aggregation has the ability and potential to cause gastrointestinal impairments. The cell type affected by aberrant protein accumulation in the GIT (enteric neurons) is post-mitotic as neurons from the CNS. This allows for a similar aggregation time of pathological aSyn species in both cell-types which is presumably years in PD patients. Although aSyn was also detected in erythrocytes (Barbour et al., 2008; Tian et al., 2019), these cells are short lived and might not display the same aggregation mechanism as long-lived neuronal cells. In colon samples the influence of dysbiosis can also be evaluated as the colon forms the interface to most commensal or pathologic bacteria.

In recent years, a better understanding of cellular processes involved in aSyn processing has helped to identify specific aSyn conformers *in vitro* and *in vivo*. As brain samples and aSyn structure can only be collected and characterized post-mortem, exclusively endpoint measurements of PD can be made. Here, samples from the GIT that can be taken at different stages of the disease might also show transient forms of aSyn aggregation. To study aSyn structure and mechanistic of aggregation, there are in principle three sources for aSyn species: (i) protein isolated from patients/animals/cells, (ii) *in vitro* aggregated aSyn conformers and (iii) amplified aSyn species from patient samples by utilizing a protein-misfolding cyclic amplification assay (PMCA) (Paciotti et al., 2018), which was initially established for prion protein analysis (Saborio et al., 2001).

The use of recombinant aSyn monomers purified from *E. coli* (Huang et al., 2005) enables studying and inducing aSyn fibril formation in a very controlled and clean environment. For this, different protocols can be applied, however, many involve constant agitation (120–1,000 rpm) for different timeframes in different buffer systems (Narkiewicz et al., 2014; Candelise et al., 2020). It was also described, that the addition of a single glass or PTFE (poly tera-flour-ethylene) bead enhances the formation of aSyn fibrils (Buell et al., 2014; Narkiewicz et al., 2014). Using transmission electron microscopy and single particle analysis helped to produce near atomic resolution structures of such an aSyn fibril (Guerrero-Ferreira et al., 2018; Li et al., 2018). This fibril consists of aSyn dimers that form an antiparallel β-sheet at the contact site with the core part ranging from amino acid 50–57. Stacking of these dimers results in the formation of amyloid fibrils that report with a pitch of 239 nm and a width of 10 nm (compared to 5 nm for an aSyn proto-fibril). A high resolution (1.4Å) structure of the NAC core domain (forms the interface of both aSyn monomers in a fibril) determined by micro electron-diffraction electron microscopy reveals the molecular interface and shows that the two aSyn monomers are not within one plane (Rodriguez et al., 2015). They are shifted upward/downward by 2.4Å. Two aSyn monomers are stacked in a distance of 4.8Å. There were also structures described for aSyn monomers carrying single amino acid exchanges also found in PD patients (Guerrero-Ferreira et al., 2019). The main question is of course, how well do these fibrils represent structures that form in patients’ brains or GIT as agitation at 1,000 rpm is not physiological. ‘Natural’ aSyn aggregates can be characterized from PD patient samples, as shown in a study analyzing aSyn fibrils derived from the CSF (Shahnawaz et al., 2020). To obtain these fibrils, CSF was taken from PD patients and the pathological aSyn species were amplified by PMCA. For this, small amounts of patient material were used as a seed and a large surplus of monomeric aSyn was added and samples were agitated to induce attachment of monomeric aSyn to the pathological aggregates adopting their conformation [PMCA; (Paciotti et al., 2018)]. Applying negative stain, cryo-tomography on the resulting conformers, revealed fibrils with a pitch of ∼260 nm and a width of 9 nm for PD patients (Shahnawaz et al., 2020). Both aSyn fibrils, recombinantly produced and amplified from patient CSF, show a similar pitch and width, and therefore, at least at the present resolution, recombinantly generated fibrils might resemble a pathological form present in PD patients. Interestingly, a different fibrillary architecture was found for MSA patients, where the cellular source of aggregated aSyn is not neuronal, but stems from oligodendrocytes (Shahnawaz et al., 2020). Hence, the cellular environment seems to influence the aggregation pattern and fibrillary structure of aSyn significantly (Candelise et al., 2020). Thus, high-resolution structural comparison of GIT- and CNS-derived aSyn conformers could help to better understand the role of the gut-brain axis in PD.

Other methods to study aSyn oligomerization and amyloid formation within GIT samples could imply fluorescent complementation assays (Herrera et al., 2012) or intercalating dyes, like Thioflavin (Vassar and Culling, 1959; Hashimoto et al., 1998; Wordehoff and Hoyer, 2018). Moreover, analysis of density and stability of different purified aSyn strains, e.g., from gastro-intestinal samples, proteinase-K, SDS or formic acid treatment could be applied (Takeda et al., 1998; Lashuel et al., 2013). The proteinase-K serine protease exerts endo- and exopeptidase activity and after aggregation of aSyn some of the cleavage sites are inaccessible for the protease resulting in an incomplete digestion, which can be visualized using coomassie stained SDS-PAGE (Cremades et al., 2012; Zunke et al., 2018). For different amyloid aSyn species, structure-specific antibodies have been raised over the last years that could be very useful for a better understanding of aSyn conformation within the GIT and disease pathology (for a comprehensive list see Harsanyiova et al., 2020). Native dot blot analysis enables the detection of folded/aggregated protein species and might help to identify conformations in patient samples of the GIT (or other sources) that are absent in controls. Characterization of GIT-derived aSyn from different PD stages by biochemical (conformation-specific aSyn antibodies) and structural (PMCA with subsequent TEM analysis) analyses as above mentioned might help to identify aggregation pathways in patients. This understanding will help to identify and characterize clinically relevant aSyn aggregates and serve as a basis to develop recombinant/cellular/animal models that can be utilized in pre-clinical intervention studies as discussed in the following paragraph.

## Implication for Therapeutic Strategies in PD

Recent years brought increasing evidence that the reciprocal connection between gut and brain may have a decisive influence on symptomatic treatment. On the one hand, it became evident that dopaminergic medication, especially Levodopa, used to improve motor function, did not improve and instead sometimes even worsens gastrointestinal symptoms such as constipation (Schaeffer and Berg, 2017). On the other hand, gastrointestinal dysfunction may affect the bioavailability and efficacy of Levodopa and therefore has a direct effect on motor function. Evidence in this respect has been seen for impaired gastric emptying (Muller et al., 2006; Doi et al., 2012) and small intestinal bacterial overgrowth as an expected result from impaired motility of the small intestine (Gabrielli et al., 2011; Fasano et al., 2013; Tan et al., 2014).

However, apart from symptomatic therapy, the gastrointestinal system might also be an important target for future disease-modifying treatment strategies. Of high interest is the possibility of influencing the microbiome in the gut (Figure 1). An increasing number of studies indicates that dysbiosis in the gastrointestinal system may play a crucial role for the pathogenesis of PD by promoting intestinal permeability, gastrointestinal inflammation and aSyn aggregation and propagation (Lubomski et al., 2019). Nutrition-based components, such as probiotics, might be able to alter enteral dysbiosis as part of pathology in PD very early in the disease. Moreover, the concept of a gut-brain route of aSyn pathology, may provide great opportunities to intervene in the earliest phase of formation and propagation of aSyn. Several compounds to modulate aSyn accumulation, aggregation and propagation are currently being investigated (Deeg et al., 2015; Wrasidlo et al., 2016; Jankovic et al., 2018). However, clinical studies still have two major short comings: first, the compound is administered in the clinical stages of the disease, in which the synucleinopathy has already wide spread and second, there is a lack of sensitive outcome parameters to verify treatment effects, as they are still mainly limited to clinical symptoms. The fairly easy accessibility of the gastrointestinal system, e.g., for biopsy studies to detect and quantify aSyn, and the development of imaging techniques to visualize gastrointestinal function and pathology, may therefore not only be of significant importance to detect individuals in the earliest phase of the disease, but also to evaluate treatment effects of disease-modifying therapies.

## Questioning the Gut-Brain Axis in PD – Weaknesses and Challenges of the Hypothesis

Although the above-mentioned points argue in favor of a gut-brain route as pathological basis in PD, this hypothesis is still subject of controversial debate, following contradictory results of clinical, pathological and animal studies [reviewed in Lionnet et al. (2018); Scheperjans et al. (2018); Leclair-Visonneau et al. (2020)]. In this respect, it is especially important to critically review the pathological findings of Braak et al., which were an important trigger for many following studies investigating the gut as primary starting point of pathology in PD. However, it must be noted that a variety of following autopsy studies could not confirm the proposed caudo-to-rostral propagation and showed that aSyn pathology in the CNS is quite often present without the occurrence of aSyn in the ENS or the vagal nerve (Jellinger, 2019). Equally, the above-mentioned studies investigating the association of vagotomy and future risk of PD were questioned by following studies, which could not find a lower PD risk in individuals receiving vagotomy (Tysnes et al., 2015; Liu et al., 2017). Additionally, the hypothesis of an exclusively caudo-rostral aSyn dissemination is questioned by both pathological findings and clinical presentation of Dementia with Lewy bodies (DLB). There is increasing consensus in the scientific community that PD and DLB belong to the same disease spectrum (Kosaka, 2014), whereby the early occurrence of cognitive dysfunction together with necortical/limbic pathology in DLB (preceding pathology in the SN) point to a rostral to caudal spread of the disease.

The alternative hypothesis of a central to peripheral spread of a-syn pathology has also been reinforced by results from animal studies, showing for example a transmission of aSyn from the midbrain via the vagus nerve into the stomach, following a CNS to PNS route (Ulusoy et al., 2017). Moreover, a recent study could show that injection and consecutive overexpression of adeno-associated aSyn in the SN lead to neuronal loss and functional alterations in the ENS, even without detectable spreading of the exogenous aSyn to the gut (O’Donovan et al., 2020). Moreover, in this study changes in the microbiome followed aSyn pathology in the SN, questioning the role of the microbiome in etiology of PD. In fact, although many studies confirmed alterations of the microbiome in PD patients, the results have to be interpreted with caution regarding potential confounders in already manifested clinical PD, such as dopaminergic medication and impaired gastrointestinal motility. More studies in prodromal cohorts and longitudinal observations are still warranted to clarify the role of the microbiome (Keshavarzian et al., 2020).

Taken together, the gut-brain hypothesis is challenged by a variety of studies in favor for a brain-to-gut transmission of aSyn pathology in PD. However, how can these two competing hypotheses be brought together? One possible way is to acknowledge possible subtypes in PD, with different ways of aSyn propagation, following either a *PNS-first or CNS-first* route, as proposed by Borghammer and Van Den Berge (2019). Another possible explanation was given by the *Threshold theory* from Engelender et al., proposing the parallel occurrence of pathology in the CNS and PNS (Engelender and Isacson, 2017). Either way, it remains of high importance to further elucidate the interaction of gastrointestinal dysfunction with aSyn formation and propagation to understand the role of the gastrointestinal system for the pathophysiology in PD.

## Conclusion

In the past years, progress has been made in understanding PD as a disease with many faces and one of these faces are alterations in the GIT homeostasis. As patient material from the CNS is limited to post-mortem samples, other sources have to be exploited. Here, samples from the GIT that contain enteric neurons might be of paramount importance. They can be taken during colonoscopy from the same patient at different stages of the disease. Enteric neurons in these samples constitute a post-mitotic neuronal cell population and with a direct interface to microbiota they might also show differences in patients with dysbiosis (Figure 1). Especially the identification of early biomarkers for the prodromal phase of PD is highly desired and these markers can only come from a non-CNS source. As GIT symptoms such as constipation often manifest years before the appearance of cardinal motor symptoms, enteric neurons might be a good cellular source for *in vivo* aggregated aSyn conformers. However, clear data to separate patient and control individuals is still missing. Importantly, amplification of pathological aSyn forms (PMCA) can generate aSyn conformers suitable for structure determination (and maybe antibody generation) to provide a better understanding of aSyn aggregation in patients. Demonstrating these aggregates in patients could well be circled back into clinical practice and might help to better define disease stages. In the future, a close collaboration between different clinical disciplines (e.g., neurology, gastroenterology, and radiology) and basic researchers (biochemists, structural biologist) will help to better understand PD on the macroscopic clinical and microscopic/biochemical level and hopefully enable new approaches toward clinical intervention studies.

## Author Contributions

All authors listed have made a substantial, direct and intellectual contribution to the work, and approved it for publication. All authors wrote the manuscript and approved its final version for publication.

## Conflict of Interest

The authors declare that the research was conducted in the absence of any commercial or financial relationships that could be construed as a potential conflict of interest.
